# Breeding in winter wheat (*Triticum aestivum* L.) can be further progressed by targeting previously neglected competitive traits

**DOI:** 10.3389/fpls.2025.1490483

**Published:** 2025-03-19

**Authors:** Annette Manntschke, Lina Hempel, Andries Temme, Marcin Reumann, Tsu-Wei Chen

**Affiliations:** ^1^ Group of Intensive Plant Food Systems, Albrecht Daniel Thaer-Institute, Faculty of Life Sciences, Humboldt Universität zu Berlin, Berlin, Germany; ^2^ Plant Breeding, Wageningen University & Research, Wageningen, Netherlands

**Keywords:** plant-plant interaction, phenotypic plasticity, canopy productivity, breeding progress, plant-plant competition, intergenotypic competition in plant, planting density

## Abstract

Breeders work to adapt winter wheat genotypes for high planting densities to pursue sustainable intensification and maximize canopy productivity. Although the effects of plant-plant competition at high planting density have been extensively reported, the quantitative relationship between competitiveness and plant performance remains unclear. In this study, we introduced a shoot competitiveness index (SCI) to quantify the competitiveness of genotypes and examined the dynamics of nine competitiveness-related traits in 200 winter wheat genotypes grown in heterogeneous canopies at two planting densities. Higher planting densities increased shoot length but reduced biomass, tiller numbers, and leaf mass per area (LMA), with trait plasticity showing at least 41% variation between genotypes. Surprisingly, genotypes with higher LMA at low density exhibited greater decreases under high density, challenging expectations from game theory. Regression analysis identified tiller number, LMA, and shoot length as key traits influencing performance under high density. Contrary to our hypothesis, early competitiveness did not guarantee sustained performance, revealing the dynamic nature of plant-plant competition. Our evaluation of breeding progress across the panel revealed a declining trend in SCI (R² = 0.61), aligning with the breeding objective of reducing plant height to reduce individual competitiveness and increase the plant-plant cooperation. The absence of historical trends in functional traits and their plasticities, such as tiller number and LMA, suggests their potential for designing ideal trait-plasticity for plant-plant cooperation and further crop improvement.

## 1 Introduction

A central objective in agronomy and breeding is the sustainable intensification of cereal crop production to enhance yield per unit area. This has driven the adaptation of maize and winter wheat varieties to high planting densities (Lollato et al., 2019; Perez et al., 2019; Bastos et al., 2020; Lacasa et al., 2022; Tombeur et al., 2022). High planting densities can optimize resource use, improve nutrient efficiency, promote early canopy closure (Perez et al., 2019; Pao et al., 2023), suppress weeds, and boost seed production (Weiner et al., 2010; Postma et al., 2021; Wheeldon et al., 2021). However, increased density also raises challenges, such as higher disease pressure, greater insect susceptibility, lodging, and elevated soil water use, which can negatively impact yield, grain quality (Reynolds et al., 2009; Weiner et al., 2017; Lollato et al., 2019) and importantly, increased plant-plant interactions (Weiner, 1990; Subrahmaniam et al., 2018). This results in the “constant final yield” concept (Weiner, 1990), which indicates that while biomass increases with density, it eventually plateaus, rendering extremely high densities impractical due to diminishing returns and increased costs (Bastos et al., 2020; Postma et al., 2021).

In the context of plant-plant interactions, increased planting density would be beneficial if the plants maximize plant-plant cooperation (commensal or reciprocal helping) and minimize plant-plant competition (competitive, selfish or altruism). Plant-plant competition is undesirable in cropping systems since competing individuals suffer from investment costs for resource capture and therefore reduce their potential productivity (Subrahmaniam et al., 2018). Using principles from game theory, theoretical ecologists have used conceptual models to study the physiological strategy of plants under inter- and intra-specific competitions (Schieving and Poorter, 1999; Vermeulen, 2015; Bongers et al., 2019). Game theory predicts that a homogenous canopy with one genotype having thicker leaves (high leaf mass per area, LMA) can be invaded by a second genotype which develops “cheap” leaves (low LMA, low construction costs) for light interception (Schieving and Poorter, 1999). Therefore, selection tends to favor individuals with low LMA, implying that an evolutionarily stable strategy results in a suboptimal canopy carbon gain. In other words, selection pressure in a competitive environment, e.g. high plant density in the agricultural systems, might favour a genotype using strategies suboptimal for its potential performance and productivity under monoculture condition (Reynolds et al., 2009; Fischer and Rebetzke, 2018). It is important to note that LMA is influenced not only by the plant’s genotypic strategy for plant-plant interactions but also by environmental conditions. For example, in resource-rich environments with ample light and CO_2_, plants tend to develop higher LMA. However, their response varies under different stress conditions—for instance, increased LMA under low temperatures or low water availability (Poorter et al., 2010). This adds another layer of complexity to the environmental effects on plant-plant interactions. The discrepancy in advantageous strategies in competitive, heterogeneous conditions implies that a breeder might select, especially in early generations (e.g. F2 to F4) where each individual plant is genetically different, genotypes having advantages in inter-genotypic competition for resource acquisition rather than genotypes with high potential in homogeneous canopies (Fasoula, 1990; Fischer and Rebetzke, 2018). For example, it has been demonstrated that high-yielding genotype exhibited lower competitiveness for radiation but compensated with greater radiation use efficiency (Cossani and Sadras, 2021). Plant height, an obvious trait related to plant-plant competition, has been intensively considered for in the context of plant breeding (Voss-Fels et al., 2019; Snowdon et al., 2021; Lacasa et al., 2022). Conversely, less obvious traits related to plant-plant competition, e.g. low LMA, might have been neglected by plant breeders and genotypes with cooperative behaviors in homogeneous canopies might have been discarded during the breeding process due to their low competitiveness with other genotypes. This implies that plant breeders, who select intensively in their fields for high individual yield, might be in the wrong direction and obtain the cultivars suboptimal for homogeneous population (Weiner, 2019; Maurer and Pillen, 2021). Therefore, it is rational to hypothesize that less obvious traits related to plant-plant interactions, such as LMA, have not yet been considered in the breeding history. Additionally, it is also important to notice the difference between plant vigor and competitiveness. Plant vigor refers to robust growth and health under ideal conditions, characterized by rapid growth and high biomass, e.g. the growth behavior under low planting density. Competitiveness, however, is a plant’s ability to secure resources and thrive among neighbor plants, often involving resource acquisition and interference with neighbors.

Many functional traits of plants exhibit plasticity in response to planting density, which significantly influences the degree of plant-plant interactions. This phenotypic plasticity is a reaction for increasing individual competitiveness for total light capture per plant (Kiaer et al., 2013; Weiner, 2019; Zhu et al., 2019; Postma et al., 2021). While numerous studies have documented systematic changes in plant traits throughout breeding history in recent years (Perez et al., 2019; Voss-Fels et al., 2019; Lichthardt et al., 2020; Welcker et al., 2022), there has been less focus on variations in phenotypic plasticity in response to environmental factors and planting density (Nimmo et al., 2023; Matesanz and Milla, 2018). Phenotypic plasticity enables a genotype to adapt to varying environmental conditions without genetic changes and there is increasing evidence for the importance of phenotypic plasticity in resource capture (Grogan et al., 2016; Nielsen and Papaj, 2022). Recent approaches suggest considering plasticity in multiple traits to gain a comprehensive understanding of a genotype’s adaptive capacity (Kikuchi et al., 2017; Nielsen and Papaj, 2022). Therefore, it is important to study multiple traits related to plant-plant competition and their plasticity in response to planting density.

In this study, we aim to unravel the complex relationships between functional traits, their phenotypic plasticity, and their effects on plant-plant competition within canopies of high planting density. Utilizing a panel of 200 winter wheat genotypes from Germany, registered between 1966 and 2016, we examined nine functional traits and their plasticity in response to varying planting densities. We hypothesize that: 1) there are significant genotypic variations in functional traits related to plant-plant competition; 2) there are genotypic differences in the plasticity of these traits; 3) plant vigor and competitiveness are distinct traits; 4) competitiveness is positively correlated with performance under heterogeneous canopy with high planting density and can be used to predict shoot biomass in the later developmental stage; and 5) traits related to plant-plant competition have changed over breeding history. Our analysis centered on how these traits exhibit plasticity under different planting densities, with the goal of elucidating their implications for wheat breeding and production.

## 2 Materials and methods

### 2.1 Plant material and experimental design

To investigate cultivar-specific responses to intergenotypic shoot competition, we grew 200 winter wheat genotypes in two density treatments in a greenhouse experiment at Humboldt Universität zu Berlin, Germany. The genotypes used are a curated collection of historical and modern German winter wheat cultivars with registration dates between 1966 and 2016, as well as a few other European and exotic cultivars to increase diversity (**Supplementary Table S1**). This panel was extensively studied in field trials, so that yield-related data is available (Voss-Fels et al., 2019; Lichthardt et al., 2020; Sabir et al., 2023; Wang et al., 2025).

In January 2022, seeds were germinated in seedling trays and grown for four weeks. Each seedling was then transplanted into a customized plastic cuboid pot (22 cm x 7 cm x 3 cm) containing 235 g of substrate (Klasmann-Deilmann, Geeste, Germany). These pots were organized into heterogeneous multi-genotype canopies (**Supplementary Figure S1**). Two treatments were established: a low planting density canopy (T1, 83 plants/m²) and a high planting density canopy (T2, 333 plants m^-2^), with one plant per genotype. In typical winter wheat fields in Germany, a planting density of 320–380 seeds/m² is common, so the competitive pressure among shoots in T2 is similar to field conditions, while T1 plants experience minimal plant-plant interactions. The position of each cultivar within a canopy was randomized, and border plants were added in T2 (**Supplementary Figure S1**) to reduce lateral light interceptions that reduce competition pressure (Chen et al., 2019). Both treatments were replicated four times in a split-plot design. Additionally, T2 was repeated with five more replicates to perform destructive measurements at two developmental stages. In total, 2600 individual plants were grown and measured. Temperatures in the greenhouse were recorded and appropriate for vernalization during seedling stages (**Supplementary Figure S2**). Sufficient amounts of nutrients and water were available in the substrate and no additional fertilizer was applied during the experiment. Plants were watered when needed, as was the application of pest controls.

### 2.2 Data collection

To quantify shoot competitiveness index (SCI) and understand how traits are associated with SCI, destructive measurements were conducted in T1 and T2 approximately 16 weeks after sowing at the booting stage (referred to as H1). To test whether the obtained SCI can be used to predict biomass in T2 at a later developmental stage, the second set of T2 was harvested four weeks later at anthesis (referred to as H2). In H1 and H2, nine functional traits were measured: 1) shoot length, 2) stage of development, 3) number of living tiller, 4) number of dead tillers, 5) number of leaves on main stem, 6) percentage of senescence of leaves on main stem, 7) total dry shoot biomass, 8) leaf area for the youngest fully developed leaves on the main stem and their 9) dry weigh. As these traits influence the performance of plants by affecting growth, reproduction and survival, they were considered as surrogates for competitiveness.

Shoot length was measured as the maximum length of the plant from the base of stem at soil height to the tip of the most extended leaf. It is important to note that shoot length, which includes both stem and leaf length, was used as a measure here because it was infeasible to measure the traditional plant height (from the base to the highest point) of individual plants in a dense, heterogeneous canopy. Developmental stage was determined by the BBCH scale, which allows a categorization of plant developmental stages based on observable characteristics (Meier, 2001). Living and dead tillers were counted, whereas dead tillers included the aborted and aborting tillers that were expected to die within the next few days. The sum of living and dead tillers resulted in the number of total tillers. The total number of leaves consisted of all leaves on the main stem of the plant, including both living and dead leaves. To assess leaf senescence on the main stem, all leaves from the bottom that were not fully green were counted. If the last wilting or yellowing leaf was still partially green, we noted its degree of wilting by adjusting the decimal point. The number of living leaves was then determined by subtracting the number of senescent leaves from the total leaf count. Leaf area was measured on the youngest fully developed leaf of the main stem using a portable leaf area meter (CI-203, CID-Bioscience, USA) and individual leaves were oven dried at 60°C for 48 hours and weighed to determine the dry mass and leaf mass per area (LMA, g m^-2^). Total shoot biomass was weighed after oven drying at 60°C for 48 hours and refers to the dry weight of the entire aboveground plant material.

### 2.3 Data analyses

For each trait, a linear mixed-effects model to account for the split-plot design was fitted by *lmer* function in *lme4* R-package (Bates et al., 2015):


$$y_{ijk}=\;\mu +\alpha_{i}+\beta_{j}+\gamma_{ij}+b_{k}+\;\epsilon_{ijk}$$

where the trait value *y* of the *i* ^th^ cultivar in the *j* ^th^ treatment is modeled with the overall mean value *μ* and the fixed effects of genotype (*α*_i_) and treatment (*β*_j_), as well as their interaction γ_ij_. The random factor *b* considered the *k* ^th^ treatment-genotype replication. *ϵ*_ijk_ was the residual errors.

Based on the model, marginal means (EMMs) of each trait was calculated, genotype and treatment and *post-hoc* pairwise comparisons using *emmeans* function in *emmeans* R-package (Searle et al., 1980; Lenth, 2017). The significance of fixed effects and their interactions were assessed using the Wald`s χ^2^ test with the *anova* function from the *car* package (Fox et al., 2019). Two-way ANOVA for each of the analyzed traits was performed. All further analyses were performed with EMMs of traits.

To quantify the proportional change in trait values due to the density treatments, we defined plasticity (*P*) of a functional trait *X* as:


$$P_{X,i}=\;\frac{X{\;}_{T2,\;i\;}-\;X{\;}_{T1,i}}{X_{\;T1,i}}$$

where for each genotype *i*, the difference of EMMs between treatments T1 and T2 is normalized to the trait EMM in T1. This standardized measure is widely used to compare the magnitudes of plasticity across genotypes and traits (Kikuchi et al., 2017; Arnold et al., 2019; Laitinen and Nikoloski, 2019).

By comparing the ability for resource capture of a plant with its neighbor in a multi-genotype canopy, the competitiveness of the plant can be quantified (Chen et al., 2019). This concept is adopted here to calculate a shoot competitiveness index (SCI):


$$SCI_{i}=\frac{SBM_{T2,i}}{\bar{SBM_{T2}}}-\frac{SBM_{T1,i}}{\bar{SBM_{T1}}}$$

where SBM is the shoot dry biomass of *i* ^th^ genotype and $\bar{\text{S}}\bar{\text{B}}\bar{\text{M}}$ is the average shoot dry biomass per plant of the whole canopy in the treatments T1 and T2, respectively. Normalization to the average response in each treatment compare the ability for resource capture of a genotype with the whole canopy in both planting density. Positive SCI values indicate above-average competitiveness in T2, while negative SCI values signify a disadvantage in dry biomass production under high planting density. Please note that reduced competitiveness does not necessarily imply cooperation. Later, we discuss cooperation in the context of plants adjusting their traits to be less selfish, such as reducing shade-avoidance responses or modifying LMA. To investigate the associations between SCI and functional traits, simple linear regression and different models of multiple linear regressions were conducted and the ability of each regression model to explain the variation in the dependent variables were compared.

To investigated how breeding history affected the functional traits, plasticity and SCI estimated in this study, breeding progress was estimated using a sliding-widow approach (Lichthardt et al., 2020). In short, ten genotypes were averaged in each sliding window, starting from the oldest to the most recent, and progressed with a step size of two. The breeding progress was determined by performing a linear regression on the resulting means, using the slope’s inclination. For this analysis, only genotypes registered for conventional agriculture in Germany were included (see Voss-Fels et al., 2019 and **Supplementary Table S1**), while others were excluded.

## 3 Results

### 3.1 Planting density affects investment trade-off for light harvesting and photosynthetic capacity at early plant development

**Figure 1:**
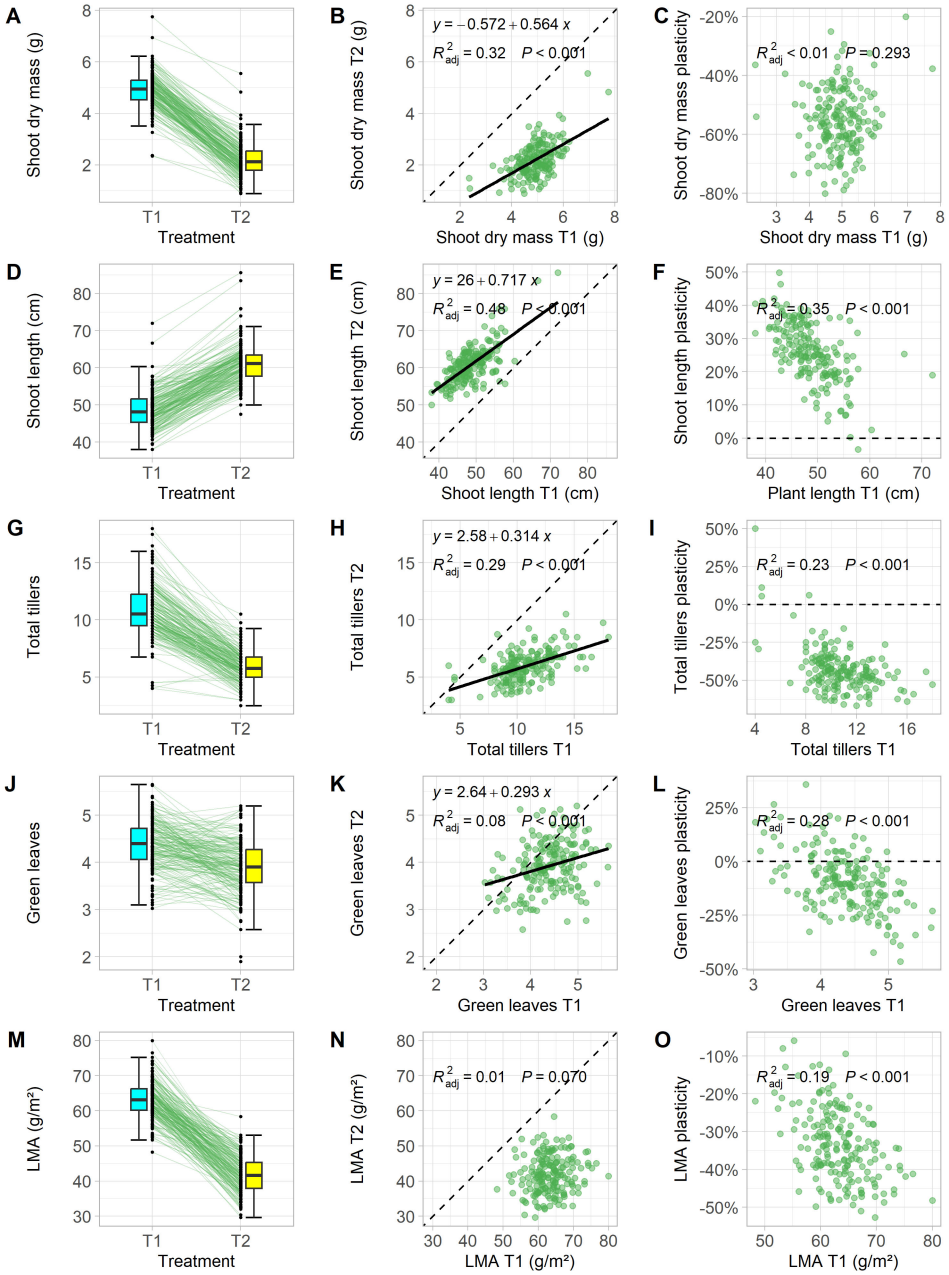
Effects of planting density on functional traits in winter wheat. Each data point represents the estimated marginal means of a genotype that were grown in low (T1) and high (T2) planting density at the booting stage. Plasticity was calculated as the percentage deviation of T2 trait values from T1. Traits shown include shoot biomass **(A–C)**, shoot length **(D–F)**, total tiller number **(G–I)**, total green leaves on the main stem **(J–L)**, and leaf mass per area [LMA, **(M–O)**]. Boxplots of trait distributions in T1 and T2, with solid lines showing genotype responses **(A, D, G, J, M)** and scatter plots of trait values in T1 vs. T2, with fitted linear regression lines, formulas, adjusted R², and p-values, with dashed lines indicating 1:1 relationship **(B, E, H, K, N)**. Comparison between plasticity and T1 trait values, with fitted linear regression lines, formulas, adjusted R², and p-values, with dashed lines marking zero plasticity **(C, F, I, L, O)**.

Analysis of variance (ANOVA) revealed significant effects of density treatments (low density: T1 and high density: T2) on leaf area (LA), leaf mass per area (LMA), shoot length (SL), shoot dry mass (SDM), total tiller number (TT), and the number of dead tillers (DT) at the booting stage (**Table 1**). For example, shoot dry mass in T2 was reduced by 20% to 80% compared to T1 (**Figure 1A**), and leaf mass per area decreased by 6% to 53% among genotypes (**Figure 1M**). Two genotypes showed reduced shoot length (SL) under T2 (**Figure 1D**), indicating their incapability to grow under increased competitive pressure.

**Table 1:** Effects of planting density on winter wheat at booting stage. Median trait values are shown for low denisty (T1) and high density (T2) treatments with ranges in parentheses.

Trait (unit)	T1		T2		T	G	T x G	Plasticity	
Leaf area (cm^2^)	20.19	(11.60, 33.25)	29.92	(16.05, 55.63)	***	***	***	51%	(-0.08, 1.30)
Senescence (leaf)	1.43	(0.55, 2.60)	1.84	(0.67, 2.88)	n.s.	**	**	28%	(-0.53, 2.82)
Shoot length (cm^2^)	48.12	(38.00, 72.00)	61.12	(47.50, 85.62)	***	***	**	25%	(-0.03, 0.50)
Total leaves (leaf)	5.75	(4.00, 8.00)	5.75	(3.00, 7.25)	n.s.	***	*	0%	(-0.31, 0.58)
BBCH stage (-)	34.50	(30.25, 58.50)	33.75	(30.00, 55.50)	n.s.	***	***	-2%	(-0.31, 0.11)
Leaf mass (g leaf^-1^)	0.13	(0.07, 0.20)	0.12	(0.08, 0.28)	n.s.	***	***	-3%	(-0.42, 0.73)
Green leaves (leaf)	4.40	(3.02, 5.65)	3.90	(1.90, 5.20)	n.s.	***	*	-10%	(-0.47, 0.36)
LMA (g m^-2^)	63.12	(48.28, 80.01)	41.62	(29.66, 58.39)	***	***	***	-34%	(-0.53, -0.06)
Tiller alive (tiller)	4.75	(2.50, 15.25)	3.00	(1.50, 7.00)	n.s.	***	***	-38%	(-0.75, 0.57)
Total tiller (tiller)	10.50	(4.00, 18.00)	5.75	(2.50, 10.50)	***	***	***	-44%	(-0.67, 0.50)
Tiller dead (tiller)	5.50	(0.00, 11.00)	3.00	(0.00, 5.50)	*	***	***	-49%	(-0.87, 5e+13)
Shoot dry mass (g)	4.94	(2.35, 7.75)	2.12	(0.89, 5.55)	***	***	***	-56%	(-0.80, -0.20)

Results of a two-way ANOVA are displayed for genotype (G), treatment (T) and their interaction (T x G) with asterisks (*p< 0.05; **p< 0.01; ***p< 0.001; n.s., not significant). The median plasticity as percentual deviation of T2 values from T1 is shown with ranges in parentheses.

High planting density led to a median plasticity increase of 51% in LA and 25% in SL (**Table 1**, **Figures 1B, C, E, F**). Both traits are considered ‘selfish’ because they enhance the plant’s ability to shade neighboring plants and increase competitiveness for light interception. In contrast, LMA, TT, and DT showed negative responses to high planting density, indicating a trade-off in resource allocation by reducing the canopy size (TT, **Figures 1H, I**) and photosynthetic capacity per leaf area (LMA, **Figures 1N, O**). ANOVA results for all 12 traits showed significant genotypic differences and genotype-treatment interactions (**Table 1**). This highlights the varied plastic responses among traits and among genotypes, suggesting genotype-specific mechanisms that optimize the cost-benefit trade-off caused by shoot competition. Understanding the performance relationships of individual genotypes in T1 and T2 is therefore crucial.

**Figure 2:**
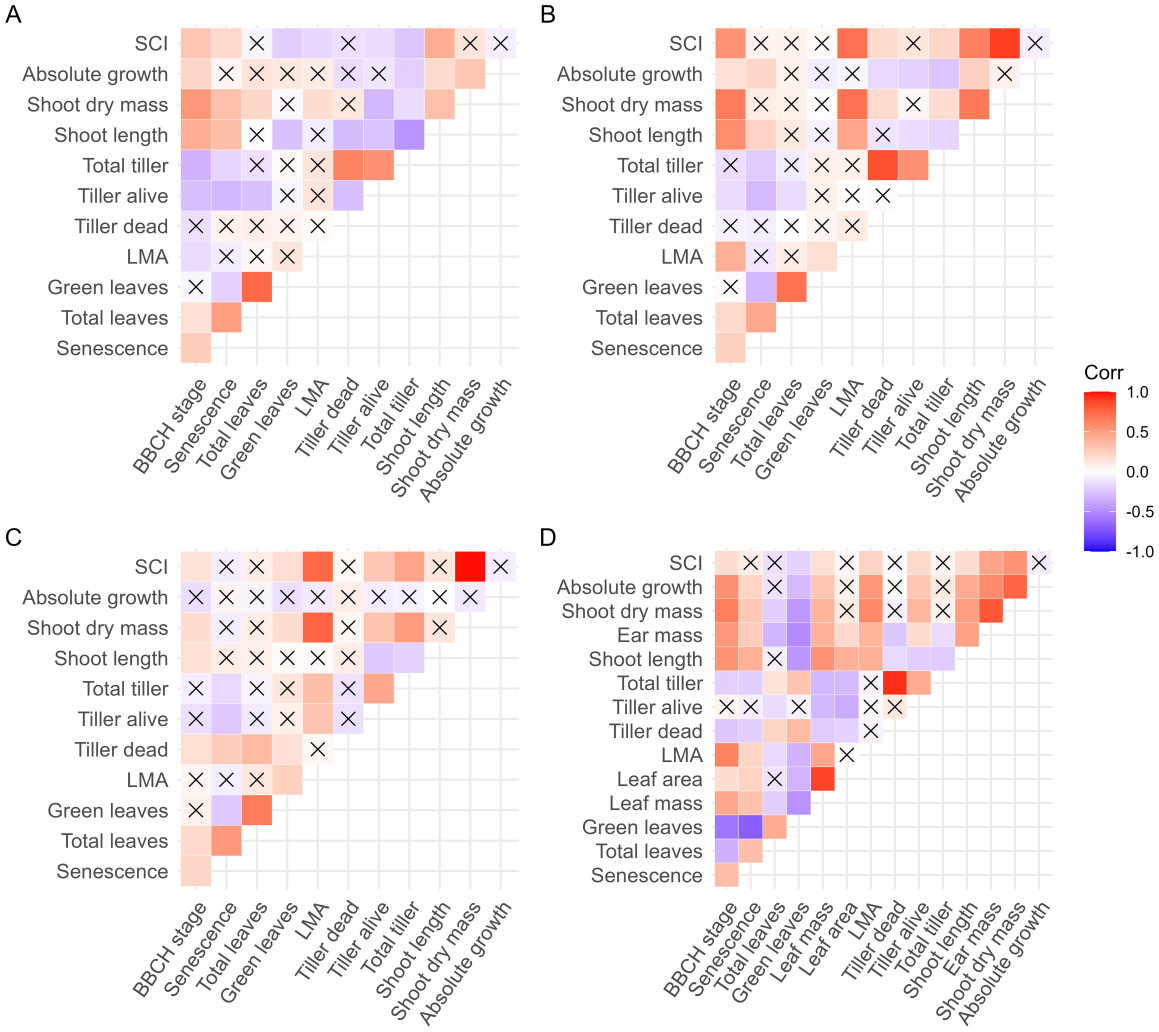
Pearson correlation coefficients between functional traits and shoot competitiveness index (SCI). **(A)** Trait values at low density at booting stage; **(B)** trait values at high density at booting stage; **(C)** plasticity of traits at booting stage; and **(D)** trait values at high density at anthesis. The correlation coefficient was calculated between pairs of traits in the 200 studied genotypes. The absolute growth was defined as the difference in shoot dry mass between booting stage and anthesis. X indicates insignificant correlation.

In treatment T1, where plant-plant interactions were minimal, SDM reflected the vigor of a genotype. Although one might expect more vigorous plants to show higher SDM in high density, only 32% of the variation in SDM in T2 could be explained by vigor alone, as shown by a linear regression model (**Figure 1B**). Remarkably, plasticity of SDM was the only trait analyzed that did not correlate performance in T1 (**Figure 1C**), suggesting a physiological independence between plant vigor and competitiveness under high density. Total tiller number (TT), which was strongly correlated with SDM (**Figure 2**), also decreased with planting density. However, 23% of the variation in plasticity for TT could be explained by TT values in T1. Genotypes with a low number of tillers in T1 either showed only a slight reduction in tiller number or even an increase in T2 (**Figure 1I**).

The number of green leaves on the main stem (GL) represent the outcome after the trade-off between density-induced leaf senescence and light harvesting. Interestingly, this trade-off, compared with LMA, is less affected by the competition pressure (**Figures 1J, M**). Under competitive conditions, maintaining leaf greenness and having a lower leaf LMA are physiologically different strategies to optimizing light capture and photosynthesis at plant level. It is interesting to observe low correlation between GL in T1 and T2 (R^2^ = 0.08, **Figure 1K**) and no correlation between LMA in the two treatments (**Figure 1N**), but moderately correlation between GL in T1 and its plasticity (R^2^ = 0.28, **Figure 1L**) and between LMA in T1 and its plasticity (R^2^ = 0.19, **Figure 1O**). This underscores another interplaying trade-off between the resource capture and resource allocation under plant-plant competition.

### 3.2 Competitive genotypes are more plastic in traits-related to plant-plant competition

To quantify the competitiveness of a genotype, the relative performance of the genotype in the whole panel between low and high planting density was compared. Although there is a high coefficient of determination between the competitiveness index (SCI) and relative plasticity of shoot dry mass in response to planting density (R^2^ = 0.97, **Supplementary Figure S3**), as SCI values deviate further from zero, the residuals to this relationship increases, indicating that SCI would correct the biased of extreme plastic responses of shoot dry mass.

It is important to emphasize that plant vigor, measured as shoot dry mass under low planting density, did not correlate with SCI (**Figure 2A**). To investigate how functional traits influenced plant vigor, shoot dry mass, and SCI, we visualized correlation matrices between these measures and among the functional traits (**Figure 2**). This visualization suggested that total tiller number (TT), leaf mass per area (LMA), and shoot length (SL) were most strongly associated with SCI and shoot biomass. Therefore, they were further used in multiple regression analysis to provide a comprehensive overview of their contributions to SCI.

Plant vigor was poorly explained by TT, LMA and PL, with R² values ranging from 0.01 to 0.15. In contrast, genotypic performance in shoot dry mass under high planting density was 62% explained by the positive effects of TT, LMA, and PL, similar to the results for dry biomass plasticity (49%) and SCI (56%, **Table 2**). Interestingly, while PL was consistently significant across models, its plasticity was significant only in explaining the SCI, asserting the differences between SCI and the plasticity of shoot biomass. SCI was slightly influenced by traits values under low-density (R² = 0.18) but significantly by high-density trait values (R² = 0.56) and plasticity (R² = 0.45). All coefficients were significant and positive, indicating greater tiller numbers, longer shoot length, and surprisingly higher leaf mass per area, enhanced competitiveness.

**Table 2:** Multiple regression models for predicting shoot biomass under low (T1) and high (T2) planting density (SBM_T1_ and SBM_T2_, respectively), plasticity of shoot biomass (SBM_Plast_), and shoot competitiveness index (SCI) using tiller number (TT), leaf mass per area (LMA) and shoot length (SL) measured at booting stage.

Model			α		β		γ		R²	
$SBM_{T1}$	=	$\text{α}*TT_{Plast}+\text{β}*LMA_{Plast}+\text{γ}*SL_{Plast}$	-0.443	n.s.	1.024	*	-0.342	n.s.	0.01
$SBM_{T1}$	=	$\text{α}*TT_{T1}+\text{β}*LMA_{T1}+\text{γ}*SL_{T1}$	-0.003	n.s.	0.026	n*	0.047	n**	0.14
$SBM_{T1}$	=	$\text{α}*TT_{T2}+\text{β}*LMA_{T2}+\text{γ}*SL_{T2}$	-0.017	n.s.	0.014	n	0.042	n**	0.15
$SBM_{T2}$	=	$\text{α}*TT_{Plast}+\text{β}*LMA_{Plast}+\text{γ}*SL_{Plast}$	0.877	n*	4.111	n**	0.479	n.s.	0.48
$SBM_{T2}$	=	$\text{α}*TT_{T1}+\text{β}*LMA_{T1}+\text{γ}*SL_{T1}$	-0.006	n.s.	-0.002	n.s.	0.069	n**	0.2
$SBM_{T2}$	=	$\text{α}*TT_{T2}+\text{β}*LMA_{T2}+\text{γ}*SL_{T2}$	0.083	n**	0.058	n**	0.062	n**	0.62
$SBM_{Plast}$	=	$\text{α}*TT_{Plast}+\text{β}*LMA_{Plast}+\text{γ}*SL_{Plast}$	0.26	n**	0.714	n**	0.138	n.s.	0.56
$SBM_{Plast}$	=	$\text{α}*TT_{T1}+\text{β}*LMA_{T1}+\text{γ}*SL_{T1}$	-0.002	n.s.	-0.003	n.s.	0.008	n**	0.1
$SBM_{Plast}$	=	$\text{α}*TT_{T2}+\text{β}*LMA_{T2}+\text{γ}*SL_{T2}$	0.017	n**	0.01	n**	0.007	n**	0.49
SCI	=	$\text{α}*TT_{Plast}+\text{β}*LMA_{Plast}+\text{γ}*SL_{Plast}$	0.626	n**	1.02	n**	0.549	n**	0.45
SCI	=	$\text{α}*TT_{T1}+\text{β}*LMA_{T1}+\text{γ}*SL_{T1}$	-0.002	n.s.	-0.007	n	0.02	n**	0.18
SCI	=	$\text{α}*TT_{T2}+\text{β}*LMA_{T2}+\text{γ}*SL_{T2}$	0.052	n**	0.011	n**	0.028	n**	0.56

The coefficients and their significance are shown (*p< 0.05; **p< 0.01; ***p< 0.001; n.s., not significant), together with the adjusted R² of each model.

**Figure 3:**
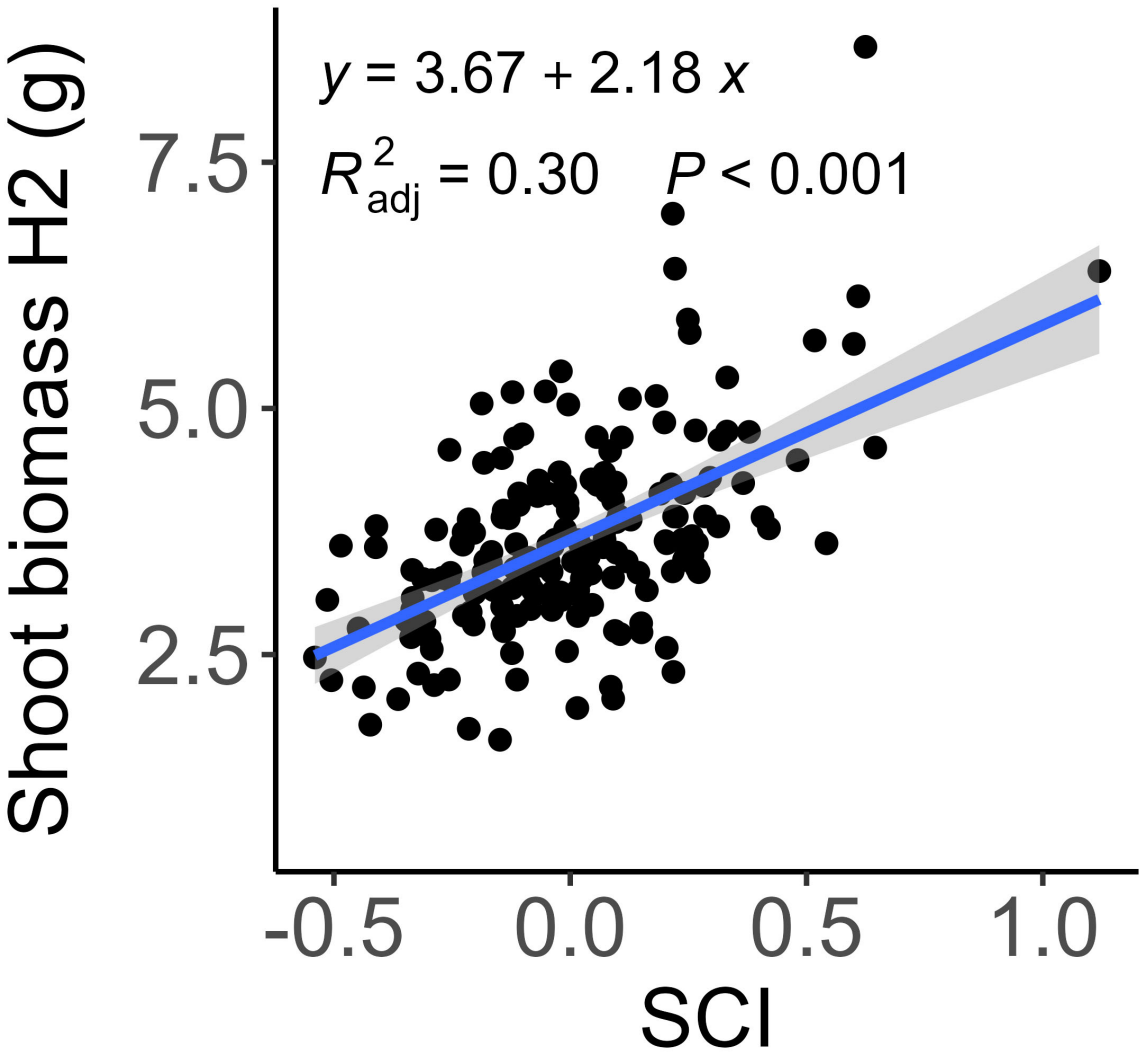
Relationship between shoot biomass at anthesis (H2) and shoot competitiveness index (SCI) at booting. Each point represents marginal mean of a genotype. The regression is shown as solid blue line. A SCI greater than 0 indicates higher competitiveness compared to neighboring plants.

Interestingly, SCI was not associated with the growth in shoot dry mass between the booting stage and anthesis (**Figure 2**) and accounted for only 30% of the variation in shoot dry mass at anthesis (**Figure 3**). This finding suggests a distinction between competitiveness and vigor or indicates that SCI may change dynamically across developmental stages. Therefore, we further examined how well the traits measured at the booting stage explained plant growth between booting and anthesis, as well as the total shoot biomass at anthesis by multiple linear regression. Using shoot biomass in high density provided a higher explanatory power (R² = 0.50) than that in low density (R² = 0.32, **Table 3**). Including SCI slightly increased the explanatory power (R² = 0.54), but unexpectedly, its negative coefficient suggests that higher competitiveness at the booting stage led to lower shoot biomass. This result is biologically not meaningful and likely reflects the poor explanatory power of the measured traits for growth between booting and anthesis (R²< 0.10, **Table 3**; **Figure 2**). Ultimately, shoot biomass at booting, total tiller number, and shoot length were the best predictors of shoot biomass at anthesis (R² = 0.55).

**Table 3:** Multiple regression models for predicting shoot biomass at anthesis (SBM_H2T2_) and absolute growth between booting and anthesis (AG) under high planting density (T2) using shoot dry biomass (SBM) shoot competitiveness index (SCI) and tiller number (TT) and shoot length (SL).

Model			α		β		γ		R²	
$SBM_{H2\;T2}$	=	$\text{α}*SBM_{H1\;T2}+\text{β}*SCI$	17.445	***	-19.397	***			0.54
$SBM_{H2\;T2}$	=	$\text{α}*SBM_{H1\;T2}$	107.233	***					0.50
$SBM_{H2\;T2}$	=	$\text{α}*SBM_{H1\;T1}$	0.886	***					0.32
AG	=	α∗SCI	-0.183	n.s.					0.00
AG	=	$\text{α}*TT_{H1\;T1}+\text{β}*SL_{H1\;T1}$	-0.058	*	0.013	n.s.			0.05
AG	=	$\text{α}*TT_{H1\;T2}+\text{β}*SL_{H1\;T2}$	-0.125	**	0.0253	*			0.09
$SBM_{H2,\;T2}$	=	$\text{α}*SBM_{H1\;T2}+\;\text{β}*TT_{H1\;T2}+\text{γ}*SL_{H1\;T2}$	0.935	***	-0.116	**	0.032	*	0.55

The subscripts denote measurement from low (T1) or high (T2) planting density at booting stage (H1) or anthesis (H2). The coefficients and their significance are shown (*p< 0.05; **p< 0.01; ***p< 0.001; n.s., not significant), together with the adjusted R² of each model.

### 3.3 Shoot competitiveness is reduced in the breeding history, but not all competitiveness-related traits were improved

**Figure 4:**
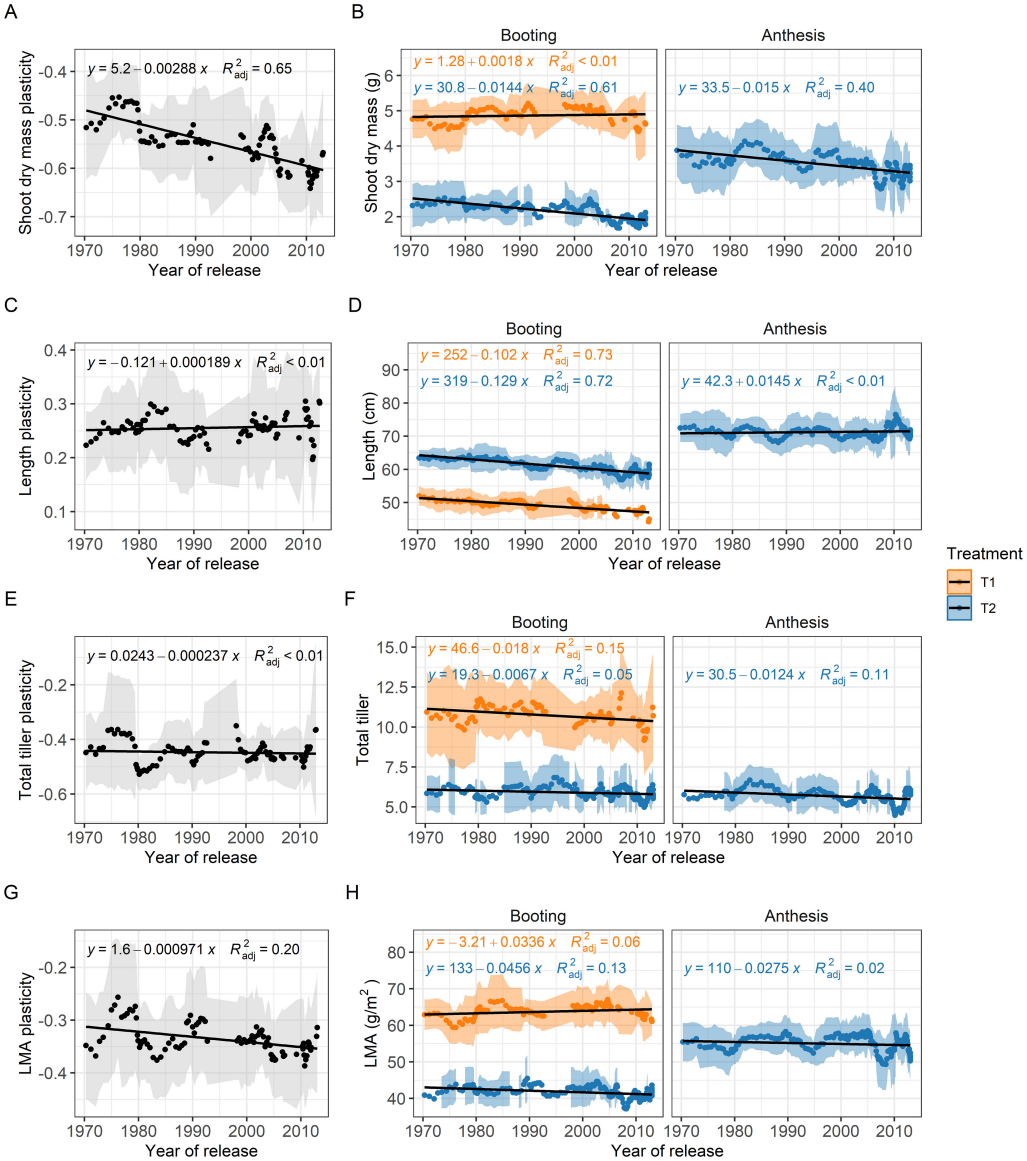
Breeding progress of shoot biomass **(B)**, shoot length **(D)**, total tiller **(F)**, leaf mass per area (LMA, **H**) and their plasticity **(A, C, E, G)** between low (T1) and high (T2) planting density. Using a sliding-window approach, each data point represents the mean values of a subset group of 10 genotypes, with the shaded area indicating the standard deviation. The black line represents the linear regression with the formula and the adjusted R² reflecting absolute breeding progress.

To evaluate the influence of breeding history on competitiveness, traits and plasticity, we conducted a sliding-window analysis. Notably, the shoot competition index (SCI), which indicates genotypes’ ability to compete in high-density environments, showed a significant decline over the years (**Figure 4**). The strong trend, with an R² value of 0.61, highlights the substantial reduction in competitiveness, as evidenced by the regression line crossing the zero line.

To further assess the breeding progress and associated changes in our wheat genotypes, we analyzed the plasticity and trait values of key functional traits identified in the multiple-linear regression analyses. This revealed a more negative plasticity of shoot biomass (R² = 0.61) over the years (**Figure 4A**), due to the findings that shoot biomass showed no distinct trend at low planting density and contrastingly a consistent decline at high planting density (slope = -0.0144 g per year; **Figure 4B**). The plasticity of shoot length and total tiller number exhibited considerable fluctuations, with minimal overall changes (R²< 0.01, **Figures 4C, E**), suggesting that plasticity in these traits has not been systematically selected for in breeding. In contrast, reduced plasticity of LMA (R² = 0.17, **Figure 4G**) suggests a slight trend towards a decrease in high planting density in more recently released genotypes, an indication of plant-plant cooperation. While shoot length decreased slightly over the years at booting stage (**Figure 4D**), genotypic values for total tiller number fluctuated, irrespective of treatment or harvest date, indicating a lack of consistent breeding impact on this trait (**Figure 4E**), similar to that in LMA (**Figure 4G**).

## 4 Discussion

Recent approaches emphasize considering plasticity in multiple traits for a comprehensive understanding of plant responses to density (Kikuchi et al., 2017; Nielsen and Papaj, 2022). We use this concept here to explore the complex relationships between nine functional traits, phenotypic plasticity, and plant-plant competition at high planting density. Using 200 winter wheat genotypes from Germany, we further examined the breeding history of phenotypic plasticity and its implications for breeding wheat. We would like to highlight two key aspects of this study. First, because the root system of each plant was isolated, plant-plant interactions were limited to the shoots. Effects of root interactions will be an interesting topic for future studies. Second, since neighboring plants in our experiments belonged to different genotypes, the canopies were heterogeneous, and all plant-plant interactions were intergenotypic, with some interpretations extended to monoculture performance.

### 4.1 Difference between inter- and intra-genotypic competitions

In conventional monocultures, plant-plant interaction is symmetrical, with evenly spaced plants of the same genotype and developmental stage (Fischer and Rebetzke, 2018; Lollato et al., 2019; Postma et al., 2021). In contrast, heterogeneous canopies—found in varietal mixtures, intercropping, organic farming using genetic populations, high-throughput phenotyping platforms, and the current study—present an asymmetrical scenario of plant-plant interaction (Chen et al., 2019; Gaudio et al., 2019; Tombeur et al., 2022; Dubs et al., 2023). While the effects of planting density on monocultures are well-documented, its impact on competitiveness and performance in heterogeneous canopies remains less understood (Litrico and Violle, 2015; Subrahmaniam et al., 2018; Beaugendre et al., 2022). Therefore, predicting the best-performing genotypes in such heterogeneous canopy is well-known challenging, even though some correlations between phenotypic traits and mixing effects have been reported. However, we are still unable to generalize robust and mechanistic rules for predicting productivity in heterogeneous canopy, e.g. varietal mixtures (Montazeaud et al., 2022; Dubs et al., 2023). This is reflected in our results, where the functional traits with the highest significance (total tiller number and shoot length) could not accurately predict absolute growth, regardless of density treatments (**Table 3**). Additionally, while differences in developmental stage could influence competitiveness, they were not significant in our study. These suggest that growth could be a highly state-specific trait, as recent literature has shown for Arabidopsis, where temporal variation in marker-trait associations for relative growth rates was detected within a growth period of only seven days (Meyer et al., 2021). Similar findings were previously made regarding wheat tiller expression at different quantitative trait loci during growth stages (Ren et al., 2018).

**Figure 5:**
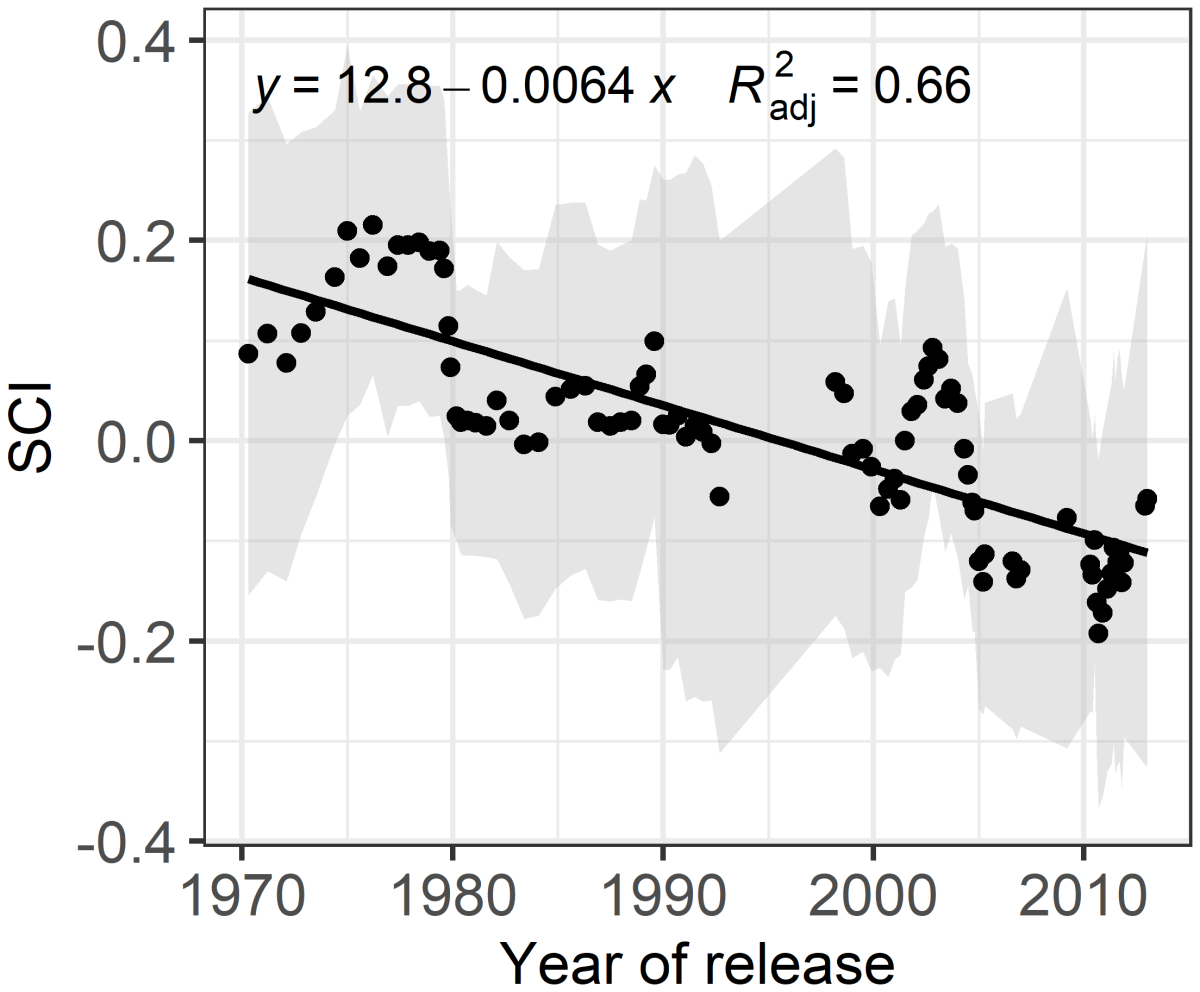
Breeding progress of shoot competitiveness index (SCI). Using a sliding-window approach, each data point represents the mean SCI of a subset group of 10 genotypes, with the shaded area indicating the standard deviation. The black line represents the linear regression with the formula and the adjusted R² reflecting absolute breeding progress.

It has been proposed that competitiveness of a plant in a heterogenous canopy can be quantified by comparing its resource capture capacity to that of neighboring plants (Chen et al., 2019). However, this method requires integrating high-throughput phenotyping platforms with computational pipelines and scientific workflow systems (Pradal et al., 2017; Artzet et al., 2019), which are not available in every lab. Therefore, we adapted this concept and proposed the shoot competitiveness index (SCI), which can be quantified in any lab and is specifically suited for heterogeneous canopies. Using this index, we show clear evidence that shoot competitiveness has decreased over breeding history (**Figure 5**), while plant vigor remains unchanged (**Figure 4B**). This aligns with the broader breeding objective to reduce plant height, minimize individual competitiveness (Colombo et al., 2022) and modify canopy architecture to optimize light distribution suitable for homogeneous canopy (Perez et al., 2019; Lacasa et al., 2022). Additionally, this panel of genotypes has been extensively studied under homogeneous canopy and field conditions, showing that modern cultivars are more productive than the older ones (Voss-Fels et al., 2019). The poorer performance of modern cultivars in heterogeneous canopies (**Figure 4B**) clearly indicates that old cultivar excelling in multi-genotypic canopies may not be optimal for monoculture conditions, emphasizing the importance of considering inter-genotypic interactions in breeding strategies (Weiner et al., 2017; Chen et al., 2019) and indicating the more cooperative responses of modern cultivars.

### 4.2 Importance of phenotypic plasticity in plant-plant interactions

In general, the observed phenotypic plasticity in response to planting density largely in accordance with the literature (Kikuchi et al., 2017; Bongers et al., 2018; Subrahmaniam et al., 2018; Poorter et al., 2019; Postma et al., 2021; Tombeur et al., 2022). We observed significant genotypic differences in the phenotypic plasticity of traits related to plant-plant competition (**Table 1**). Interestingly, the lack of strong correlations among the plasticity of different functional traits (**Figure 2C**) suggests a decoupling of their physiological regulation, offering potential to optimize combination of plasticity in response to planting density and enhance plant-plant cooperation under competition pressure.

Although Chen et al. (2019) advanced the quantification of plant-plant competition, their morpho-physiological explanation of competitiveness remains less clear. Furthermore, in their analyses, highly competitive genotypes have higher biomass production than but similar leaf area to the less competitive ones, implying thicker leaves or stem of the highly competitive genotypes (higher in LMA and in stem mass per length). This contradicts the prediction of game theory suggesting that competitive genotypes should produce thinner leaves (Schieving and Poorter, 1999) but in accordance with our results (**Table 2**; **Figure 2B**). Given that LMA, among 12 environmental factors, exhibits the highest plasticity in response to light quantity (Poorter et al., 2010), our findings propose an alternative hypothesis: a highly competitive genotype may initially produce cheaper leaves with low LMA and subsequently exhibit greater plasticity in adjusting LMA to the light environment encountered post-developmentally (**Figure 2C**). This could explain the observed low plasticity in LMA (less negative plasticity) within the competitive genotype (**Figure 2C**). Since LMA and leaf area were measured from a single leaf, they may not fully reflect whole-plant variation. It will be interesting, although labor-intensive, to exam the response of LMA to competition at the whole-plant level.

The high correlation between LMA and photosynthetic capacity suggests that plasticity in LMA can be explained by the strategy of developmental and post-developmental photosynthetic acclimation (Pao et al., 2019b), which can be explained by the concept of photosynthetic protein turnover (Pao et al., 2019a). Recently, it has been demonstrated that sensitivity of photosynthetic protein synthesis rate to light (referred to as photosynthetic acclimation strategies, PAS), is especially crucial for the leaf plasticity in response to light environments (Pao et al., 2023). This suggests complex feedbacks between PAS, light gradient resulted from the canopy architecture and canopy productivity. Therefore, optimizing canopy productivity requires coordination between PAS and dynamics in canopy architecture. This coordination becomes more complex and more difficult to be achieved in a heterogeneous canopy, where PAS and architecture of each individual plant are different. The clear association between the plasticity of LMA and SCI in this study (**Table 2**) could be an indirect evidence supporting the importance of this coordination. To our knowledge, this type of plant-plant interactions has not yet been investigated and appears as a missing link towards a full understanding of the productivity of heterogeneous canopies.

### 4.3 Breeding progress in winter wheat can be further optimized by targeting previously neglected competitive traits

Yield stagnation in European wheat production since the mid-nineties (Brisson et al., 2010; Bönecke et al., 2020) highlights the need to identify traits that could further increase yield. Targeting traits related to plant-plant competition is particularly important, as competition for resources in dense planting environments reduces canopy performance (Weiner et al., 2010; Kiaer et al., 2013; Subrahmaniam et al., 2018). Reducing the negative effects of light competition at high planting densities could enhance future genetic progress (Perez et al., 2019; Colombo et al., 2022). In this study, we investigated the breeding progress of plant-plant competition by using a shoot competitiveness index and analyzing their competitiveness-related traits and their phenotypic plasticity in a set of 200 genotypes used by other researchers in our field (Lichthardt et al., 2020; Sabir et al., 2023). Our results suggest that modern genotypes exhibit more cooperative behavior, possibly a hidden factor in breeding success. Further study is needed to identify which traits contribute to plant-plant cooperation. Potential candidates include architectural traits or their plasticity, as breeding progress in maize shoot architecture has been documented (Mantilla-Perez and Salas Fernandez, 2017; Perez et al., 2019; Lacasa et al., 2022). Although it was infeasible to study architectural traits in a heterogeneous canopy of current study, it will be interesting to explore these traits and their plasticity using high-throughput phenotyping platforms in the future.

As wheat breeding programs progress, integrating insights into trait plasticity and competitiveness is crucial. Our study highlights how plasticity of functional traits affects competitiveness, yet its diverse aspects have not been targeted by breeders (**Figure 4**). Designing ideal combinations of plasticity to increase plant-plant cooperation could sustain breeding progress. Future studies in this direction are promising, as they will further unravel the intricate relationships between trait plasticity, competitiveness, and performance in high-density conditions within heterogeneous canopies, laying a foundation for continued refinement in wheat breeding programs.

## Data Availability

The original contributions presented in the study are included in the article/**Supplementary Material**. Further inquiries can be directed to the corresponding author.
