# Supplementary material for: Breeding in winter wheat (Triticum aestivum L.) can be further progressed by targeting previously neglected competitive traits
Source: Front Plant Sci. 2025 Mar 19;16:1490483. doi: 10.3389/fpls.2025.1490483 (PMC11961425; doi:10.3389/fpls.2025.1490483)
Supplement: Supplementary file 1 [file DataSheet1.pdf]

Supplementary Material

1 Supplementary Data

Supplementary Material should be uploaded separately on submission. Please include any supplementary data, figures and/or tables.

Supplementary material is not typeset so please ensure that all information is clearly presented, the appropriate caption is included in the file and not in the manuscript, and that the style conforms to the rest of the article.

2 Supplementary Figures and Tables

2.1 Supplementary Figures

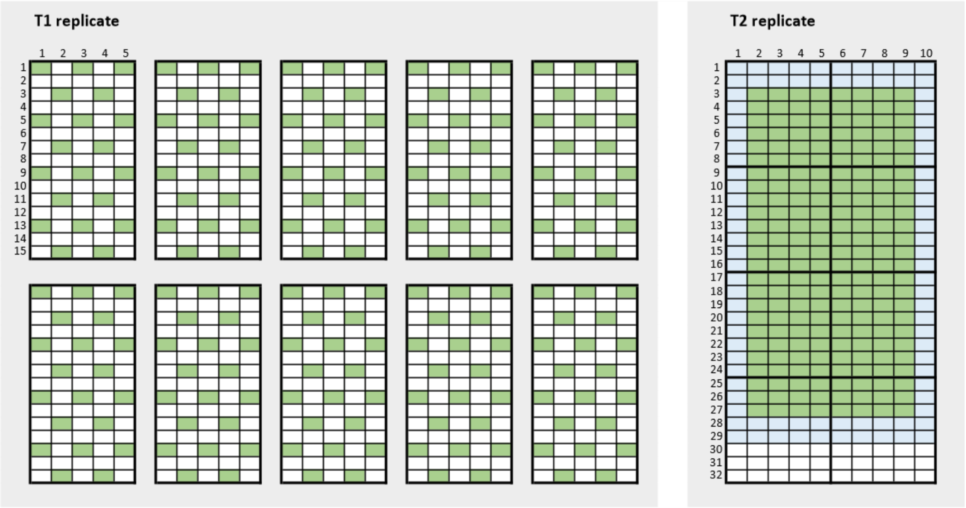

Supplementary Figure S1. Experimental design with multi-genotype canopies. Containers, depicted as thick black lines, were used to hold pots, shown as thin black lines, in both low (T1) and high (T2) planting density arrangements. Pots marked in green contain one of the 200 studied genotypes in each treatment. Pots marked in light blue indicate border plants, which consisted of a random subset of the cultivar panel, while pots without color were left empty.

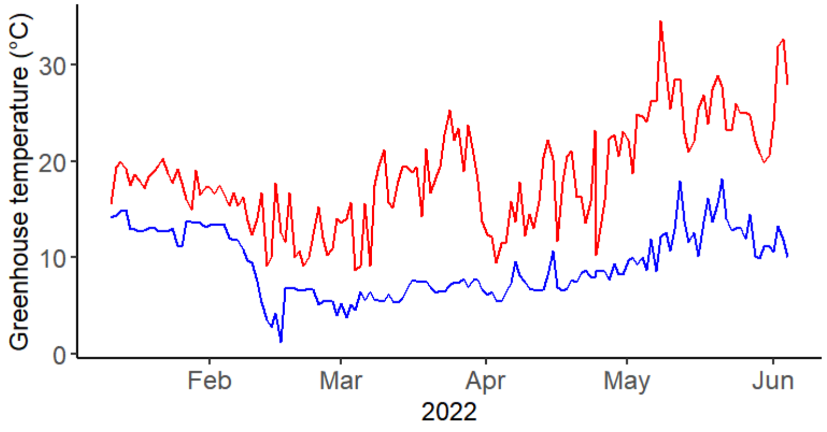

Supplementary Figure S2. Greenhouse temperature data were collected by averaging the maximum (red) and minimum (blue) temperature values recorded by five to ten sensors, which took measurements every five minutes.

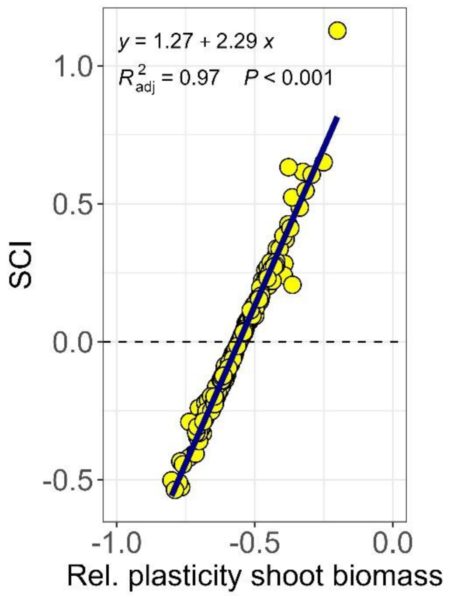

Supplementary Figure S3. Relationship between shoot competitiveness index (SCI) and relative plasticity in shoot biomass. Each circle in yellow represents one genotype and values were calculated from estimated marginal means. The regression line is shown in dark blue.
